# Antibacterial activity of Punica granatum L. and Areca nut (P.A) combined extracts against some food born pathogenic bacteria

**DOI:** 10.1016/j.sjbs.2021.10.057

**Published:** 2021-10-25

**Authors:** Neda Jam, Reza Hajimohammadi, Parvin Gharbani, Ali Mehrizad

**Affiliations:** a Department of Chemical Engineering, Ahar Branch, Islamic Azad University, Ahar, Iran; b Department of Chemistry, Ahar Branch, Islamic Azad University, Ahar, Iran; c Department of Chemistry, Tabriz Branch, Islamic Azad University, Tabriz, Iran

**Keywords:** Antibacterial, Areca nut, Combined extracts, Punica granatum L

## Abstract

The antibacterial effects of combined extracts of Punica granatum L. and Areca nut (P.A) against resistant bacteria, a gram-positive bacterium, *Staphylococcus aureus* and three gram-negative bacteria, *Escherichia coli, Salmonella,* and *Enterobacter aerogenes,* in individual and biofilm forms was studied. Antibacterial activity was studied using disk diffusion method, microbroth dilution, and microtiter plate methods. Given the disc diffusion test (Minimum Inhibitory Concentration (MIC) and Minimum Bactericidal Concentration (MBC)), the extracts had inhibitory effects on the individual forms of bacteria. However, the ethanolic extract had greater effectiveness than the methanolic extract. Generally, ethanol efficiently extracts flavonoids and their glycosides, catechol and tannins. This fact due to the ethanol polarity that is equal 0.654. The results indicated that the ability of extracts in inhibiting the formation of biofilms, destruction of biofilms, and prevention of metabolic activity of bacteria had a direct relationship with concentration and the highest inhibitory was seen on *Staphylococcus aureus* (98.98%), *Staphylococcus aureus* (94.98%), and *Enterobacter aerogenes* (88.55%). Based on the results, the P.A. combined extract can be used as an alternative combination with the ability to inhibit antibiotic-resistant bacteria in single and biofilm forms.

## 1 Introduction

Diseases caused by eating foods contaminated with pathogenic bacteria are very important to public health and inflict heavy financial and human losses on communities. Therefore, controlling the growth of pathogenic bacteria in food is important in terms of standard quality regulations as well as public health. Today, resistance to antimicrobial drugs has become a major global problem due to the indiscriminate use of antimicrobial drugs and the formation of biofilm structures (Mohammadi et al., 2019, Sadeghian et al., 2012). A biofilm is a structure composed of a bacterial population surrounded by an exopolymer matrix and produced by bacteria. This feature allows bacteria to bind to different levels and increase innate resistance to antibiotics (Duran et al., 2012). Bacterial biofilms allow colonization and persistence on host surfaces as well as escape from phagocytosis of immune cells. Although biofilm is not a precondition for persistent infection, it represents a virulence phenotype that significantly protects bacteria from environmental factors (Kristian et al., 2004). Biofilm formation from one bacterium is relatively like another, a sequence that is summarized in four main stages: adhesion, microclone development, biofilm maturation, and dispersion. Adherence of biofilms to materials is mainly regulated by hydrophobic interactions, steric interactions, protein adhesion, electrostatic interactions, and Van der Waal forces, all of which help bacteria to adhere to the surface (Khatoon et al., 2018). Extracellular matrix production, which began in the development of microclones and continued during biofilm maturation, has adhesion function and allows bacterial communities to form a rigid structure. These extracellular matrices are complex environments composed primarily of water (97%), proteins, nucleic acids, lipids / phospholipids, and exopolysaccharide (EPS) polymers that vary by bacterial species. Interestingly, EPS plays an important role in the protective properties of the biofilm matrix. Biofilm formation can be considered as the real mechanism of bacterial resistance to antibiotics (Rasamiravaka et al., 2015)

Biofilm formation reduces the susceptibility to antimicrobial therapies and ultimately increases the cost of treating patients (Atshan et al., 2012). They are more resistant to biocides and disinfectants (Bae et al., 2012). The growth of biofilm on food processing equipment causes microbial contamination in the production process and thus reduces the shelf life of products and increases the prevalence of foodborne diseases. These biofilms contain spoilage or pathogenic microorganisms (Bryers, 2000, Palmer et al., 2007).

Biofilms are thriving in the food industry and in some cases can become a chronic problem. Therefore, much research has been done to find alternative strategies for the prevention and treatment of biofilm-based infections. It has been suggested that the best possible treatment for biofilm-based infections is to inhibit the initial binding phase.

**Fig. 1:**
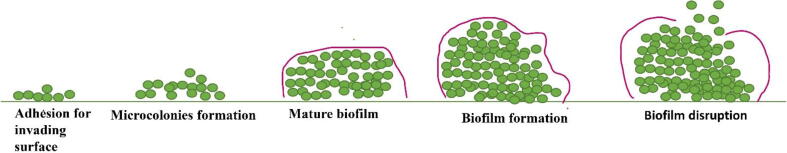
Schematic of Biofilm formation and destruction on the surface.

Bacteria are released thanks to active and/or inactive movements (swimming, crowding, contraction, gliding, gliding, and shooting). In the presence of suitable surfaces and environments, the invasion begins with the stages of adhesion and microcline formation, which leads to the development of mature biofilms in four main stages (adhesion, development of microclones, biofilm maturation and dispersion) (Khatoon et al., 2018). The programmed dispersion of microbial cells is the final stage of ecological evolution, which is achieved by the release of newly formed cells from biological materials or the separation of the constituent cells by species-specific saccharolytic enzymes. Erosion and slippage may also occur due to mechanical pressure on the biological layer, which causes peripheral cells to leave the environment uncontrollably and enter the local environment. Although the dispersed cells move again, the cells remain physiologically unique in the planktonic and biological phases; These scattered cells are very violent towards macrophages, which is a useful feature due to their main purpose of colonizing new areas. Dispersion is one of the worst stages of a biological life cycle, but it may be one of the easiest to target, resulting in the possibility of killing microbial cells (LuTheryn et al., 2020). Bacterial infections are mainly due to their ability to attack and spread through their host using different types of motility, with the release of numerous virulence agents, by creating structured biofilms lead to damage to host cells and tissues. Fig. 1 shows a summary of the formation and destruction of the biofilm on the surface (Khatoon et al., 2018).

Recently, increasing attention is being paid to antimicrobial compounds with herbal and animal origins that improve microbial and chemical control and increase food shelf life (Burt, 2004). Today, due to the widespread use of antibiotics, bacterial resistance to antibiotics has increased (Mohammadi et al., 2019). One of the reasons for the development of antibiotic-resistant bacteria is the formation of biofilms, which has become a serious problem in infections. Biofilms are organisms attached to microbial cells with strong adhesion to surfaces and are protected by an extracellular matrix consisting of exopolysaccharides, proteins, and DNA and this matrix plays a protective role of the cell against various factors that prevent the penetration of antimicrobial compounds and their proper function; As some researchers claim, the biofilm resistance of bacteria to antibiotics is thousands of times higher than the single bacterial form (Saeidi et al., 2015). While resistant bacteria have become commonplace in health care facilities, inadequate experimental treatment has been reported to increase mortality from resistant pathogens (Kang et al., 2005). With the increase in antimicrobial resistance and the emergence of new infectious agents, many natural products have been directly examined for antimicrobial activity and the ability to modulate resistance (Coutinho et al., 2009). While natural products play an important role in the development of new drugs and play an important role in the treatment and prevention of diseases, herbal antimicrobials provide the required therapeutic drugs, the advantages of which include low production costs, low side effects, no environmental problems. In herbal medicine research, synergistic evaluation between herbal medicines and common antibiotics has been used, because many diseases have complex causes and pathophysiology that require treatment with appropriate drug combinations rather than single drug therapy (Bellini et al., 2008).

Essential oils, extracts, medicinal and edible plants can be the most important sources of drugs with new antibacterial and antifungal effects due to their antimicrobial compounds (Chakraborty et al., 2014, Hassanshahian et al., 2014). Reducing the use of antibiotics, controlling microbial contamination of foods, developing technologies to improve shelf-life, eliminating undesirable pathogens, and delaying microbial spoilage, and reducing the resistance of pathogenic microorganisms by increasing cellular resistance are some benefits of these natural antimicrobials (Saeidi et al., 2014, Weinstein, 2001).

A wide range of chemicals in plants can inhibit bacterial pathogens (Medina et al., 2005). The successful determination of such biologically active compounds from plant materials largely depends on the type of solvent used in the extraction method. Organic solvents such as ethanol, acetone and methanol are often used to extract bioactive compounds. However, ethanol is the most common organic solvent used by herbal medicine manufacturers because consumers can safely use the final products (Dog, 2009).

This study investigated the synergic antimicrobial effects of pomegranate and Areca nut extracts. Pomegranate with a scientific name, Punica granatunutm L., belongs to the family Punicaceae. Pomegranate has been not only considered as a fruit, its medicinal properties, and application in the food industry have been also taken into consideration by many researchers (Wang et al., 2018). All flowers, leaves, peel, green and young stems, root bark, fruit and seed bark, and finally extract and juice of pomegranate are used therapeutically (Guerrero-Solano et al., 2020). Pomegranate is rich in bioactive compounds, mainly polyphenols, and anthocyanins. Polyphenols are very powerful antioxidants. The antioxidant properties of phenolic compounds depend on their ability to give electrons to trap free radicals by forming stable phenoxyl compounds. Alginic acid is an important chemical compound in pomegranate peel, and its phenolic nature and structure cause its strong antioxidant activity (Brighenti et al., 2017). The main phenolic and tannic compounds in pomegranate are Ellagic acid, Gallic acid, Punicalagin, Punicalin, Chlorogenic acid, etc (Fischer et al., 2011, Zarezadeh Mehrizi et al., 2017). Areca nut or Betel nut is a tree of the family Palmaceae with the scientific name “Areca catechu L.” and a synonym of “Areca faufel Gartn”. In terms of chemical composition in Areca nut, it has Isoguvocine, Arecaine, Arecaidine, Arecoline, Guvacine, Arecodin, Arecatine, and Guvacoline. Its fruit has alpha-Catechin and its seeds have Arecatine and Arecodin. Areca seeds contain about 15% tannin, about 14% fat, a little sugar, sucrose, mannan, galactan, etc (Bhat, 2016). Another laboratory study report that there are at least four alkaloids in Areca seeds, called Guvacoline, Arecoline, Arecaidine, and Guvacine, among which Guvacoline and Arecoline are the most important components that increase the natural elasticity and elasticity of the intestines, as well as the smoky movement of the intestines without damaging the respiratory and circulatory systems (Zhang et al., 2009). Most studies on the extracts of these two medicinal fruits have been done mostly on a single form of bacteria. The present study examined the antibacterial activity of the combined extract (methanolic and ethanolic) of pomegranate and Areca nut (P.A) against important pathogenic bacteria in the food industry, including Staphylococcus aureus, Escherichia coli, Salmonella, and Enterobacter aerogenes, in biofilm and single forms.

## 2 Materials and methods

### 2.1 Collection, detection, and extraction of essential oil

Wild pomegranate fruits were collected from Arasbaran forests (in fall) and approved by the Agricultural and Natural Resources Research Center, and Areca nut fruit was prepared from Hakim Razi Azar Co (It was prepared from India and was collected in Fall)*.* After washing, the fruits were dried in a freeze dryer for 24 h (Fig. 1s). Then finely ground using an electric mill (Model: A11 Basic Analytical mill; power = 160 w; rotational speed = 2000 rpm) for 10 min. and filtered in 25 mesh sieves. Dried fruits were re-dried by an oven at 40 ℃ for 24 h and were extracted. The maceration method was used for extraction which powder of each fruit was mixed with methanol and ethanol and stirred (900 rpm) in a shaker incubator at 40 ℃ during 18–24 h. After filtration, it was transferred to a rotary device to remove excess solvent. The solution was incubated at 40 ℃ for 48–72 h to dry. The powder was stored in dark glass containers at −4 ℃ until use.

### 2.2 Microorganisms and culture medium

In the present study, we used a gram-positive bacterium, *Staphylococcus aureus (ATCC 6538)*, and three gram-negative bacteria, *Escherichia coli* (ATCC 25922), *Enterobacter aerogenes* (ATCC 13048), and *Salmonella enteric* (ATCC 9270) from Pasteur Institute of Iran and were stored at 4 °C. We utilized Muller Hinton Agar media for determine Disk Diffusion, Minimum Inhibitory Concentration (MIC) and Minimum Bactericidal Concentration (MBC).

### 2.3 Examination of antimicrobial effects of plant extracts using the disk diffusion method

The antimicrobial activity of crude extract was examined using the Kirby-Bauer Disk Diffusion method. In summary, the bacteria were diluted after culture to reach a turbidity of 1.5 × 10^8^ cfu/mL. turbidity was adjusted to 0.5 McFarland’s index in phosphate buffered saline. The 6 mm blank disks were immersed inside the extract solution at a concentration of 100 mg/mL for 1 h. With the help of sterile forceps, the disks containing 100 mg/mL extract were placed in a sterile plate to dry at room temperature. 100 mg of dried extract was dissolved in 1 cc of methanol or ethanol, and then the discs were immersed in this solution for 1 h, then removed from the solution, dried at room temperature, weighed again and the absorption of the extract based on the weight difference and gravimetry were calculated. The weight of the discs was recorded before immersion in the solution. Finally, prepared disks were placed at regular distances on the medium and the plates containing bacteria were incubated at 37 °C for 24 h. Discs containing ethanol and methanol were considered as the control. The diameters of the inhibition zone of the discs were measured using a millimeter ruler (Sepehri et al., 2014).

### 2.4 Minimum inhibitory concentration (MIC) and Minimum Bactericidal concentration (MBC)

The values of MIC and MBC of P.A. combined extract were measured using the microbroth dilution method according to the European Committee for Antimicrobial Susceptibility Testing (EUCAST). In this method, 10 dilutions of ethanolic and methanolic extract solution were prepared using the sequential dilution method to determine MBC and MIC. Then 1 mL of bacterial suspension with a turbidity of 5 × 10^5^ cfu/mL was added to the Nutrient broth and incubated at 37 °C for 24 h. Bacterium along with Nutrient broth (NB) medium was considered as the control. The tubes were compared with control turbidity and the lowest concentration with an inhibited bacterial growth was considered as MIC. To determine the MBC of each extract, dilutions of MIC without visible turbidity were used. 100 mL of these dilutions were cultured on the Nutrient agar medium by a table-cloth culture method and incubated at 37 °C for 24 h. Finally, the lowest concentration at which the bacteria did not grow after incubation for 24 h at 37 °C, was considered as MBC (Rezaie Keikhaie et al., 2018).

### 2.5 Ability to form biofilms by strains and ability of the extract to prevent the biofilm formation

The ability of strains to form biofilms was examined using O'Toole and Kolter method. Biofilm formation was studied using O'Toole and Kolter method by slightly changes (Jabra-Rizk et al., 2006). First, three dilutions of extracts (6.25–25 mg/mL from ethanolic and methanolic extracts) were prepared and 100 μL of each was added to 96 well microplates. Then, 100 μL of bacterial suspension was added to the well and incubated at 37 °C for 24 h. The well with TSB and sterile water was considered as the control. After incubation, the microtiter plate was rinsed with Phosphate-Buffered Saline (PBS) three times. Then, 150 μL of methanol 96% was added to stabilize the adherent cells. After removal of methanol, 200 μL of Crystal Violet (1%)) was added to the well and incubated at 30 °C for 5 min. Finally, 160 μL of glacial acetic acid (33%) was added to the well and the light absorbance was read by ELISA Reader (Anthos 2020 USA) at λ = 630 nm and the percentage of inhibition of biofilm formation was obtained from Eq. (1) (Sandasi, 2008).

(1) $$M\%=100\times \left\{\left(A-B\right)-\left(C-D\right)\;/\;\left(A-B\right)\right\}$$

where, M% is inhibition of biofilm formation, A,B, C and D are average light absorbance of the control, culture medium, well and extract, respectively.

### 2.6 The ability of the extract in the destruction of biofilm structures

To form of biofilms, 100 μL of bacterial cultures was incubated in the Tryptic soy broth (TSB) medium in 96-well microplates at 37 °C for 24 h. After biofilm formation, the medium was slowly emptied, and the non-adhesive cells was removed by rinsing the biofilms with sterile PBS (2 times). To investigate the effect of the extract on the preformed biofilm, each extract at different concentrations of 6.25–25 mg/mL was added to wells and incubated at 37 °C for 24 h. The biofilm inhibition was analyzed by crystalline violet staining. Reduction percentage of biofilm structures at different concentrations of extracts was calculated using Eq. (1) (Ramage and López-Rib, 2005).

### 2.7 The ability of the extract on the dehydrogenase activity

First, pre-formed biofilms were rinsed with PBS, and then different concentrations (6.25–25 mg/mL) of extracts were added and incubated at 37 °C for 24 h. After incubation, 50 μL of Triphenyl Tetrazolium Chloride (TTC) was added to each well and incubated at 37 °C for 3 h. Final absorption was recorded at 630 nm and the reduction of biofilm metabolic activity in the presence of different concentrations of extracts were measured using Eq. (1) (Høiby et al., 2010).

## 3 Results

The antibiotic resistance of bacteria due to the use of antibiotics is the main cause of infection in humans and causes the emergence and spread of antibiotic-resistant bacterial strains. More than 80% of infectious bacteria in humans form biofilm structures; hence, many efforts have been made to obtain more information about the active ingredients in plants and their application in the treatment of various infections and biofilm control (Ghotaslou and Salahi, 2013, Høiby et al., 2010). The use of plant-based antimicrobials can play a valuable role in controlling the formation of biofilms and infectious diseases so that 80% of people in developed countries use herbs to treat various diseases (Nascimento et al., 2000).

### 3.1 Inhibitory effects of the combined extract on the single forms of bacteria

Even though many antibacterial and antifungal activities of plant extracts have been reported, their effectiveness against antibiotic-resistant bacteria is very low. In this regard, the present study investigated the antibacterial properties of the P.A combined extract on four species of antibiotic-resistant bacteria. Table 1 presents the mean inhibition zone diameters (mm) results of P.A. combined extracts (ethanol & methanol solvents). According to Table 1, methanolic extract showed the highest and lowest antibacterial activity against *Salmonella enteric* (21 ± 1.24) and *Escherichia coli* (8 ± 1.11), respectively. While, ethanolic extract resulted highest and lowest antibacterial activity against Staphylococcus *aureus* (20 ± 0.64 mm) and *Escherichia coli* (10 ± 1.25 mm), respectively. Also, the largest diameter of inhibition zones of ethanolic and methanolic extract of P.A. belonged to *Staphylococcus aureus* (20 ± 0.64 mm) and *Salmonella enteric* (21 ± 1.14 mm). According to Table 1, methanolic extract showed the highest and lowest antibacterial activity against Salmonella enteric (21.21.21) and Escherichia coli (8 ± 1.8), respectively.

**Table 1:** Mean inhibition zone diameters of P.A. combined extracts.

**Bacteria**	**mean inhibition zone diameters (mm)**	
	**Ethanolic**	**Methanolic**
*Staphylococcus aureus*	20 ± 0.64	9 ± 0.20
*Salmonella enteric*	18 ± 0.25	21 ± 1.24
*Enterobacter aerogenes*	12 ± 0.45	11 ± 0.15
*Escherichia coli*	10 ± 1.25	8 ± 1.1

MIC and MBC results of methanolic and ethanolic extracts of P.A. in the range of 25–50 mg/mL are given in Table 2. In the present investigation, lowest MIC value 25 ± 0.845 value was recorded for P.A. extract in ethanol against *Enterobacter aerogenes* as well as in methanol against Enterobacter aerogenes and Escherichia coli. The results show that MIC of ethanolic extract of P.A. for *Salmonella enteric* and *Enterobacter aerogenes* was 50 ± 0.21 and 25 ± 0.845 mg/mL, respectively, while it is had no effect against *Staphylococcus aureus* and *Escherichia coli* . Also, MIC results of methanolic extract of P.A. for *Staphylococcus aureus*, *Enterobacter aerogenes* and *Escherichia coli* was 50 ± 1.11, 25 ± 1.01 and 25 ± 1.21 mg/mL, respectively but it had no effect against Salmonella *enteric*.

**Table 2:** MIC and MBC of P.A. combined extracts.

Bioactivity of different extracts against pathogens				MIC/MBC	Solvents
*Escherichia coli*	*Enterobacter aerogenes*	*Salmonella enteric*	*Staphylococcus aureus*		
------	25 ± 0.845	50 ± 0.21	-----	MIC	Ethanol
50 ± 0.75	50 ± 0.54	------	25 ± 0.65	MBC	
25 ± 1.21	25 ± 1.01	------	50 ± 1.11	MIC	Methanol
50 ± 0.87	-------	25 ± 0.98	-------	MBC	

The results show that MBC of ethanolic extract of P.A. for *Staphylococcus aureus*, *Enterobacter aerogenes* and *Escherichia coli*, *were* 25 ± 0.65, 50 ± 0.54 and 50 ± 0.75, respectively but it had no effect on *Salmonella enteric*. Also, methanolic extract of P.A. for, *Salmonella enteric and Escherichia coli were* 25 ± 0.98 and 50 ± 0.87, respectively, while it had no effect on *Staphylococcus aureus* and *Enterobacter aerogenes.* MBC values for ethanol extracts were found higher than the MIC values of the extracts against microorganisms tested.

### 3.2 Inhibitory effects of P.A. Combined extract against biofilm formation

**Fig 2:**
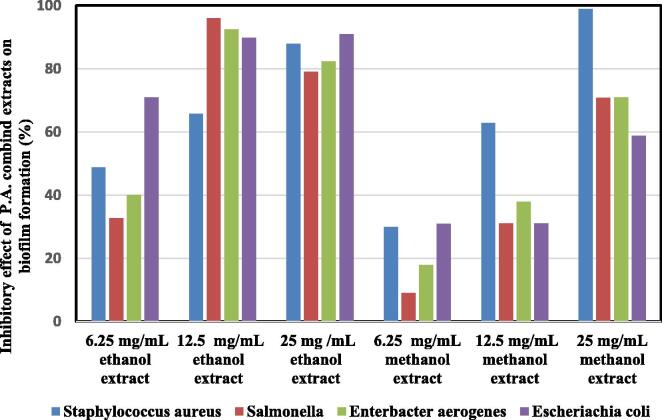
Inhibitory effect of combined extract on the formation of biofilms at various concentrations.

Fig. 2 shows the capability of different concentrations of P.A. combined extract on the formation of biofilms. Accordingly, the highest inhibition of biofilm formation was seen with methanolic extract at a concentration of 25 mg/mL on *Staphylococcus aureus* (98.8%) and the lowest inhibitory effect belonged to the same extract at a concentration of 6.25 mg/mL on *Salmonella enteric* (8.98%) strain. The P.A. combined extract had a significant effect on different bacteria in inhibiting the biofilm formation (p < 0.05).

### 3.3 Inhibitory effects of the combined extract against the destruction of the biofilm

**Fig. 3:**
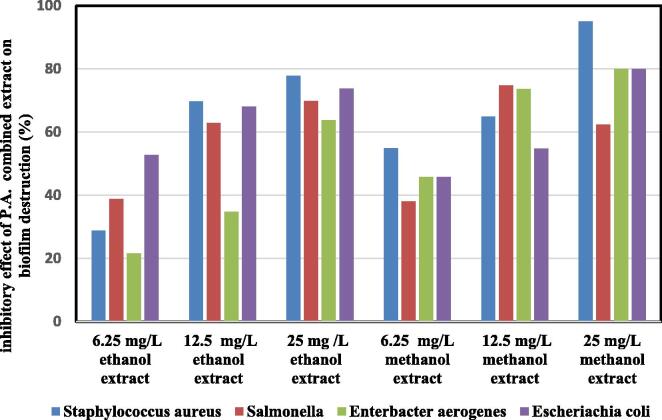
Inhibitory effect of P.A. combined extract on biofilm destruction at different concentrations.

Fig. 3 presents the capability of different concentrations of the P.A. combined extracts in the destruction of biofilm structures. The highest destruction of biofilm structures belonged to the treatment with the methanolic extract at a concentration of 25 mg/mL on the *Staphylococcus aureus* strain (94.98%), and the lowest destruction belonged to ethanolic extract at a concentration of 6.25 mg/mL (21.54%) on the *Enterobacter aerogenes* strain (21.54%). The effect of individual P.A. combined extract was significant (P < 0.05).

### 3.4 Inhibitory effects of the combined extract on dehydrogenase activity

**Fig. 4:**
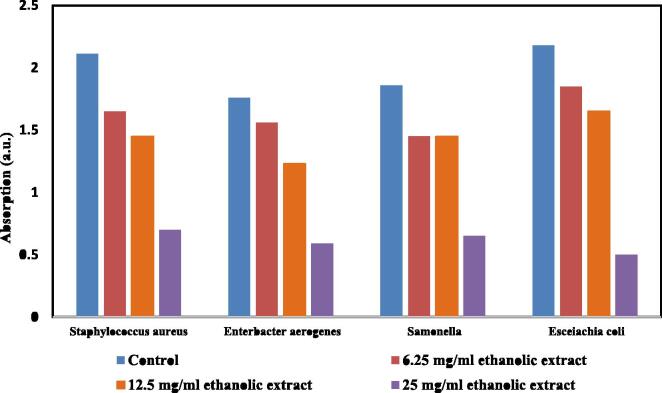
Inhibitory effects of the P.A. combined ethanolic extract on dehydrogenase activity.

**Fig. 5:**
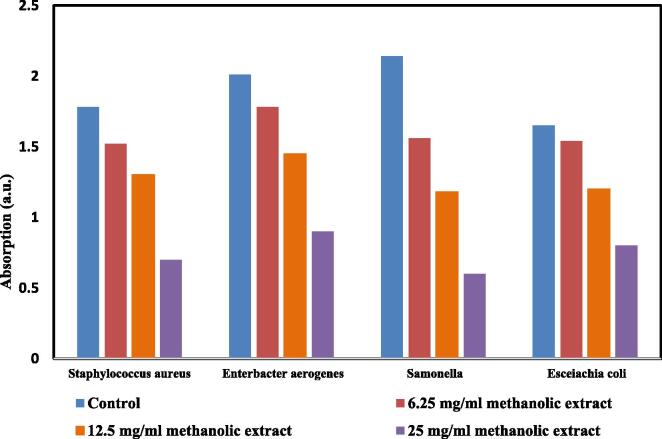
Inhibitory effects of the P.A. combined methanolic extract on dehydrogenase activity.

Fig. 4, Fig. 5 show the capability of each different concentration of the P.A. combined extract on the dehydrogenase activity of the bacteria. The highest inhibition of enzymatic activity belonged to the treatment with ethanolic and methanolic extracts at a concentration of 25 mg/mL on *Escherichia coli* (OD = 0.5) and *Salmonella enteric* (OD = 0.6). Furthermore, the lowest inhibition of enzymatic activity belonged to the treatment with ethanolic and methanolic extracts at a concentration of 6.25 mg/mL on *Escherichia coli* (OD = 1.85) and *Enterobacter aerogenes* (OD = 1.78), respectively. There was a significant effect of P.A. combined extract on dehydrogenase activity of different bacteria (P < 0.05).

## 4 Discussion

The results of the antimicrobial effects of methanolic and ethanolic extracts is revealed that both extracts had the inhibitory effect on *Staphylococcus aureus*, *Escherichia coli*, *Enterobacter aerogenes* and *Salmonella enteric.*

Results of MBC, & MIC indicated that the P.A. combined extract had a great ability to prevent the growth of antibiotic-resistant bacteria. Furthermore, values ​​obtained from MIC and MBC in a range of 25–50 mg/mL indicated that the highest inhibitory concentrations of ethanolic and methanolic extracts belonged to *Salmonella enteric* (50 ± 0.21 mg/mL) and Staphylococcus aureus (50 ± 1.11 mg/mL). Similar results confirmed the inhibitory effects of green tea extract on Methicillin-resistant *Staphylococcus aureus* (MRSA), Multi-drug resistant *Pseudomonas aeruginosa* (MDR-aruginosa), *Staphylococcus aureus*, and *Escherichia coli*. The property was generally associated with polyphenol compounds in green tea extract against gram-positive and gram-negative bacteria (Cui et al., 2012, Liaqat et al., 2016). The inhibitory effects of Teucrium polium extract on Methicillin-resistant *Staphylococcus aureus (MRSA), Staphylococcus aureus*, and *Escherichia coli* were also confirmed (Darabpour et al., 2010).

Solvent extraction is most frequently used technique for extract of plant antioxidant compounds. However, the extract yields and resulting antioxidant activities of the plant materials are strongly dependent on the nature of extracting solvent, due to the presence of different antioxidant compounds of varied chemical characteristics and polarities that may or may not be soluble in a solvent. Polar solvents are frequently employed for the recovery of polyphenols from a plant matrix. The most suitable of these solvents are (hot or cold) aqueous mixtures containing ethanol, methanol, acetone, and ethyl acetate. Methanol and ethanol have been extensively used to extract antioxidant compounds from various plants and plant-based foods (fruits, vegetables etc.) Generally, acetone is the best solvent for extracting pro anthocyanidins and tannins; ethanol efficiently extracts flavonoids and their glycosides, catechols and tannins; whereas phenolic acids and catechin were better extracted with methanol. These facts agree with polarity of the solvent used for the extraction and solubility of phenolics in them since the polarity of ethanol and methanol is 0.654 and 0.762, respectively. Ethanol is a safe and effective organic solvent to extract polyphenols from plants. Ethanol was selected as the extraction solvent since it is commonly used in the food industry in a variety of ways and are more highly stable in the human body than any other solvents since it is commonly used in the food industry in a variety of ways and are more highly stable in the human body than any other solvents. Among the different medicinal plant materials, aqueous ethanolic extract of Acacia nilotica bark offered the highest total phenolic contents (Li et al., 2020).

The difference between the effectiveness of the extract in the MBC, MIC and disk diffusion experiments is probably due to the difference in the diffusion between the extract from the discs containing it and the diffusion in the test liquid medium.

It can be concluded, the ethanolic solvent has been more effective than the methanolic in reacting with components and ingredients of the P.A. extract and due to increase the release of active substances from the plant and enhancing concentrations of these substances in ethanolic extract compared to methanolic extract is more effective. Areca nuts have many compounds including fatty acids (myristic acid, palmitic acid, stearic acid, oleic acid, oleic acid, decanoic acid and hexadecenoic acid), alkaloids (arecoline, guavcine, guvacoline), polyphenols (Catechins, epicatechin, leucocyanidin and procyanidins) as well as several minerals and vitamins. The main antibacterial compounds in areca nuts are the fatty acids in areca nuts, namely myristic acid and oleic acid, and procyanidins, which work by inhibiting the enzyme glucosyltransferase. It has also been reported that the tannic acid in Areca nuts can stop the growth of bacteria (Peng et al., 2015). Polyphenolic compounds also have antibacterial effects, but their antibacterial mechanism is not yet well understood. The pervious papers was reported the antibacterial activity of polyphenols through hydrogen bonding with intracellular enzymes or changes in cell walls and changes in cell membrane permeability(Bouarab-Chibane et al., 2019). Flavonoids, which are polyphenols, can bind to membrane-soluble proteins and inhibit energy metabolism and DNA synthesis. Polyphenols in gram-positive bacteria also change the intracellular pH of bacteria, leading to bacterial death (Cushnie and Lamb, 2011). Also, Punica granatum L. contains 17 types of amino acids, large amounts of vitamin C, calcium, iron and phosphorus, as well as small amounts of retinol, riboflavin, ferulic acid and other phenolic compounds (Ge et al., 2021). The antibacterial activity of Punica granatum L is due to its high content of polyphenols, especially flavonoids and tannins (lignin punicalagins, paeoniflorin punicalins, gallic acid and ellagic acid). The results show that these phenolic compounds show synergistic effects on various microorganisms (Fawole et al., 2012) .

The antibacterial effects of this combined extract may be due to the compounds to which the microorganisms have never been exposed (Bhat, 2016, Guerrero-Solano et al., 2020, Wang et al., 2018).

optimized content of Punica granatum L. and Areca nut over the bacterial inactivation was investigated. Optimized concentration of P.A. on the inactivation of *Staphylococcus aureus* & *Salmonella* was obtained with both ethanolic & methanolic extracts at 25 mg/L of P.A. However, optimum concentration of P.A. on inactivation of *Escherichia coli* & *Enterobacter aerogenes* pathogens was obtained at 25 mg/L of ethanolic extracts. In general, it can be concluded that increasing the concentration of the P.A. extract improves the inactivation of all studied pathogens (Fig. 4, Fig. 5). May be, Since the concentration of the P.A. extract increases, the permeability of antibacterial agents to the bacterial cell wall increased.

Plants play important roles in eliminating the biofilm formation of pathogenic bacteria. The composition of their extracts prevents the formation and development of biofilms. The present study indicated that the P.A. combined extract was effective against biofilm structure, and the inhibitory effect of the extract was directly related to concentrations. The capability of ethanolic extract to inhibit biofilm was greater than biofilm destruction or inhibition of the metabolic activity of microbial cells in the biofilm structure. We can conclude that ethanolic extract contains a molecule that is associated with bacterial biofilm formation, but this extract has little ability to overcome biofilm structure. The extracts of these plants probably prevent the formation of biofilms by affecting the adhesion and the bacterial quorum-sensing system (Agrawal, 2011).

## 5 Conclusion

Based on the results of the present study, the P.A. compound extract often had a greater ability to inhibit the biofilm formation of foodborne pathogenic bacteria. Accordingly, this extract can be used to improve the performance of antibiotics or even replace them. Among the extracts, the ethanolic extract had the highest antimicrobial activity. However, gram-negative bacteria had greater resistance to extracts than gram-positive bacteria.

## Declarations

**Ethics approval:** Not Applicable.
**Consent to participate:** All authors consent to participate in this manuscript.
**Consent for publication:** All authors consent to publish this manuscript in Saudi Journal of Biological Science.
**Availability of data and material:** Data will be available on request to corresponding or first author.
**Code availability:** Not Applicable.
**Author contributions:** Neda Jam & Reza Hajimohammadi drafted the experimental design and performed the experiments. Ali Mehrizad and Parvin Gharbani helped in data collection and data analysis. Parvin Gharbani wrote initial draft of manuscript and revised the manuscript to present form. All authors read the manuscript before communication.

## Declaration of Competing Interest

The authors declare that they have no known competing financial interests or personal relationships that could have appeared to influence the work reported in this paper.

## References

[b0005] Agrawal I. (2011). Susceptibility of bacterial biofilms against some leaf extracts. Plant Sci. Feed.

[b0010] Atshan, S.S., Nor Shamsudin, M., Sekawi, Z., Lung, L.T.T., Hamat, R.A., Karunanidhi, A., Mateg Ali, A., Ghaznavi-Rad, E., Ghasemzadeh-Moghaddam, H., Chong Seng, J.S., 2012. Prevalence of adhesion and regulation of biofilm-related genes in different clones of Staphylococcus aureus. J. Biomed. Biotechnol. 2012. 10.1155/2012/976972 PMC3372070 22701309

[b0015] Bae Y.-M., Baek S.-Y., Lee S.-Y. (2012). Resistance of pathogenic bacteria on the surface of stainless steel depending on attachment form and efficacy of chemical sanitizers. Int. J. Food Microbiol..

[b0020] Bellini M.F., Cabrioti L.N., Terezan A.P., Jordão B.Q., Ribeiro L.R., Mantovani M.S. (2008). Cytotoxicity and genotoxicity of Agaricus blazei methanolic extract fractions assessed using gene and chromosomal mutation assays. Genet. Mol. Biol..

[b0025] Bhat K.S. (2016). Action of arecanut (Areca Catechu L.) and its chewing forms on laboratory animals and its implication on human carcinogenesis-An assessment. Indian J. Arecanut. Spices Med. Plants.

[b0030] Bouarab-Chibane L., Forquet V., Lantéri P., Clément Y., Léonard-Akkari L., Oulahal N., Degraeve P., Bordes C. (2019). Antibacterial properties of polyphenols: characterization and QSAR (Quantitative structure–activity relationship) models. Front. Microbiol..

[b0035] Brighenti V., Groothuis S.F., Prencipe F.P., Amir R., Benvenuti S., Pellati F. (2017). Metabolite fingerprinting of Punica granatum L. (pomegranate) polyphenols by means of high-performance liquid chromatography with diode array and electrospray ionization-mass spectrometry detection. J. Chromatogr. a.

[b0040] Bryers J.D. (2000).

[b0045] Burt S. (2004). Essential oils: their antibacterial properties and potential applications in foods—a review. Int. J. Food Microbiol..

[b0050] Chakraborty B., Nath A., Saikia H., Sengupta M. (2014). Bactericidal activity of selected medicinal plants against multidrug resistant bacterial strains from clinical isolates. Asian Pac. J. Trop. Med..

[b0055] Coutinho H.D.M., Costa J.G.M., Lima E.O., Falcão-Silva V.S., Júnior J.P.S. (2009). Herbal therapy associated with antibiotic therapy: potentiation of the antibiotic activity against methicillin–resistant Staphylococcus aureus by Turnera ulmifolia L. BMC Complement. Altern. Med..

[b0060] Cui Y., Oh Y.J., Lim J., Youn M., Lee I., Pak H.K., Park W., Jo W., Park S. (2012). AFM study of the differential inhibitory effects of the green tea polyphenol (−)-epigallocatechin-3-gallate (EGCG) against Gram-positive and Gram-negative bacteria. Food Microbiol..

[b0065] Cushnie T.P.T., Lamb A.J. (2011). Recent advances in understanding the antibacterial properties of flavonoids. Int. J. Antimicrob. Agents.

[b0070] Darabpour E., Motamedi H., Nejad S.M.S. (2010). Antimicrobial properties of Teucrium polium against some clinical pathogens. Asian Pac. J. Trop. Med..

[b0075] Dog T.L. (2009). Smart Talk on Supplements and Botanicals: Exotic Fruits—Acai, Noni, Mangosteen, Sea Buckthorn, and Goji. Altern. Compliment. Ther..

[b0080] Duran N., Ozer B., Duran G.G., Onlen Y., Demir C. (2012). Antibiotic resistance genes & susceptibility patterns in staphylococci. Indian J. Med. Res..

[b0085] Fawole O.A., Makunga N.P., Opara U.L. (2012). Antibacterial, antioxidant and tyrosinase-inhibition activities of pomegranate fruit peel methanolic extract. BMC Complement. Altern. Med..

[b0090] Fischer U.A., Carle R., Kammerer D.R. (2011). Identification and quantification of phenolic compounds from pomegranate (Punica granatum L.) peel, mesocarp, aril and differently produced juices by HPLC-DAD–ESI/MSn. Food Chem..

[b0095] Ge, S., Duo, L., Wang, J., Yang, J., Li, Z., Tu, Y., 2021. A unique understanding of traditional medicine of pomegranate, Punica granatum L. and its current research status. J. Ethnopharmacol. 113877. 10.1016/j.jep.2021.113877 33515685

[b0100] Ghotaslou R., Salahi B. (2013). Effects of oxygen on in-vitro biofilm formation and antimicrobial resistance of Pseudomonas aeruginosae. Pharm. Sci..

[b0105] Guerrero-Solano J.A., Jaramillo-Morales O.A., Jiménez-Cabrera T., Urrutia-Hernández T.A., Chehue-Romero A., Olvera-Hernández E.G., Bautista M. (2020). Punica protopunica Balf., the Forgotten Sister of the Common Pomegranate (Punica granatum L.): Features and Medicinal Properties—A Review. Plants.

[b0110] Hassanshahian, M., Bayat, Z., Saeidi, S., Shiri, Y., 2014. Antimicrobial activity of Trachyspermum ammi essential oil against human bacterial.

[b0115] Høiby N., Bjarnsholt T., Givskov M., Molin S., Ciofu O. (2010). Antibiotic resistance of bacterial biofilms. Int. J. Antimicrob. Agents.

[b0120] Jabra-Rizk M.A., Meiller T.F., James C.E., Shirtliff M.E. (2006). Effect of farnesol on Staphylococcus aureus biofilm formation and antimicrobial susceptibility. Antimicrob. Agents Chemother..

[b0125] Kang C.-I., Kim S.-H., Park W.B., Lee K.-D., Kim H.-B., Kim E.-C., Oh M., Choe K.-W. (2005). Bloodstream infections caused by antibiotic-resistant gram-negative bacilli: risk factors for mortality and impact of inappropriate initial antimicrobial therapy on outcome. Antimicrob. Agents Chemother..

[b0130] Khatoon Z., McTiernan C.D., Suuronen E.J., Mah T.-F., Alarcon E.I. (2018). Bacterial biofilm formation on implantable devices and approaches to its treatment and prevention. Heliyon.

[b0135] Kristian S.A., Golda T., Ferracin F., Cramton S.E., Neumeister B., Peschel A., Götz F., Landmann R. (2004). The ability of biofilm formation does not influence virulence of Staphylococcus aureus and host response in a mouse tissue cage infection model. Microb. Pathog..

[b0140] Li C.-W., Chu Y.-C., Huang C.-Y., Fu S.-L., Chen J.-J. (2020). Evaluation of Antioxidant and Anti-α-glucosidase Activities of Various Solvent Extracts and Major Bioactive Components from the Seeds of Myristica fragrans. Molecules.

[b0145] Liaqat I., Pervaiz Q., Bukhsh S.J., Ahmed S.I., Jahan N. (2016). Investigation of bactericidal effects of medicinal plant extracts on clinical isolates and monitoring their biofilm forming potential. Pak Vet J.

[b0150] LuTheryn G., Glynne-Jones P., Webb J.S., Carugo D. (2020). Ultrasound-mediated therapies for the treatment of biofilms in chronic wounds: a review of present knowledge. Microb. Biotechnol..

[b0155] Medina A.L., Lucero M.E., Holguin F.O., Estell R.E., Posakony J.J., Simon J., O’Connell M.A. (2005). Composition and antimicrobial activity of Anemopsis californica leaf oil. J. Agric. Food Chem..

[b0160] Mohammadi M., Masoumipour F., Hassanshahian M., Jafarinasab T. (2019). Study the antibacterial and antibiofilm activity of Carum copticum against antibiotic-resistant bacteria in planktonic and biofilm forms. Microb. Pathog..

[b0165] Nascimento G.G.F., Locatelli J., Freitas P.C., Silva G.L. (2000). Antibacterial activity of plant extracts and phytochemicals on antibiotic-resistant bacteria. Brazilian J. Microbiol..

[b0170] Palmer J., Flint S., Brooks J. (2007). Bacterial cell attachment, the beginning of a biofilm. J. Ind. Microbiol. Biotechnol..

[b0175] Peng W., Liu Y.-J., Wu N., Sun T., He X.-Y., Gao Y.-X., Wu C.-J. (2015). Areca catechu L. (Arecaceae): A review of its traditional uses, botany, phytochemistry, pharmacology and toxicology. J. Ethnopharmacol..

[b0180] Ramage G., López-Rib J.L. (2005). Techniques for antifungal susceptibility testing of Candida albicans biofilms. Antifungal Agents. Springer.

[b0185] Rasamiravaka, T., Labtani, Q., Duez, P., El Jaziri, M., 2015. The formation of biofilms by Pseudomonas aeruginosa: a review of the natural and synthetic compounds interfering with control mechanisms. Biomed Res. Int. 2015. 10.1155/2015/759348 PMC4383298 25866808

[b0190] Rezaie Keikhaie K., Bagheri G., Hassanshahian M., Saeidi S. (2018). Antimicrobial effects of Zataria multiflora essential oils on Acinetobacter strains isolated from clinical specimens. J. Herb. Drugs (An Int. J. Med. Herbs).

[b0195] Sadeghian, I., Hassanshahian, M., Sadeghian, S., Jamali, S., 2012. Antimicrobial effects of Quercus brantii fruits on bacterial pathogens.

[b0200] Saeidi, S., Boroujeni, N.A., Ahmadi, H., Hassanshahian, M., 2015. Antibacterial activity of some plant extracts against extended-spectrum beta-lactamase producing Escherichia coli isolates. Jundishapur J. Microbiol. 8. 10.5812/jjm.15434 PMC4353063 25793093

[b0205] Saeidi S., Shiri Y., Bokaeian M., Hassanshahian M. (2014). Antibacterial Activity of Essential Oil of Sature jahortensis Against Multi-DrugResistant Bacteria. Int. J. Enteric Pathog.

[b0210] Sandasi, M., 2008. The effect of plant extracts on microbial biofilm formation and development.

[b0215] Sepehri Z., Hassanshahian M., Shahi Z., Nasiri A., Baigi S. (2014). Antibacterial effect of ethanol extract of Camellia sinensis l against Escherichia coli. Asian Pacific J. Microbiol. Res..

[b0220] Wang D., Özen C., Abu-Reidah I.M., Chigurupati S., Patra J.K., Horbanczuk J.O., Jóźwik A., Tzvetkov N.T., Uhrin P., Atanasov A.G. (2018). Vasculoprotective effects of pomegranate (Punica granatum L.). Front. Pharmacol..

[b0225] Weinstein R.A. (2001). Controlling antimicrobial resistance in hospitals: infection control and use of antibiotics. Emerg. Infect. Dis..

[b0230] Zarezadeh Mehrizi R., Emam-Djomeh Z., Shahedi M., Keramat J., Rezaei K., Loni E. (2017). Phenolic compounds and antioxidant activity of dried peel of Iranian pomegranate. J. Food Qual. Hazards Control.

[b0235] Zhang W., Li B., Han L., Zhang H. (2009). Antioxidant activities of extracts from areca (Areca catectu L.) flower, husk and seed. African. J. Biotechnol..

