# Supplementary material for: Antibacterial activity of Punica granatum L. and Areca nut (P.A) combined extracts against some food born pathogenic bacteria
Source: Saudi J Biol Sci. 2021 Oct 25;29(3):1730–6. doi: 10.1016/j.sjbs.2021.10.057 (PMC8913389; doi:10.1016/j.sjbs.2021.10.057)
Supplement: Supplementary data 1 [file mmc1.docx]

Fig. 1s shows the used Freeze dryer for dying


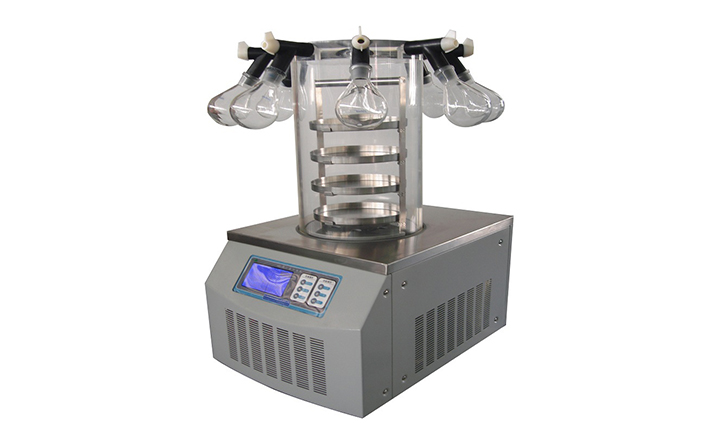


Fig. 1s. Used Freeze dryer

*Antibacterial activity of Areca Nut fruit extracts*

The antibacterial activity of different concentrations of methanolic, ethanolic and aqueous extracts of *Areca nut* fruit against selected pathogens studied and results are presented Fig. 2s.

**
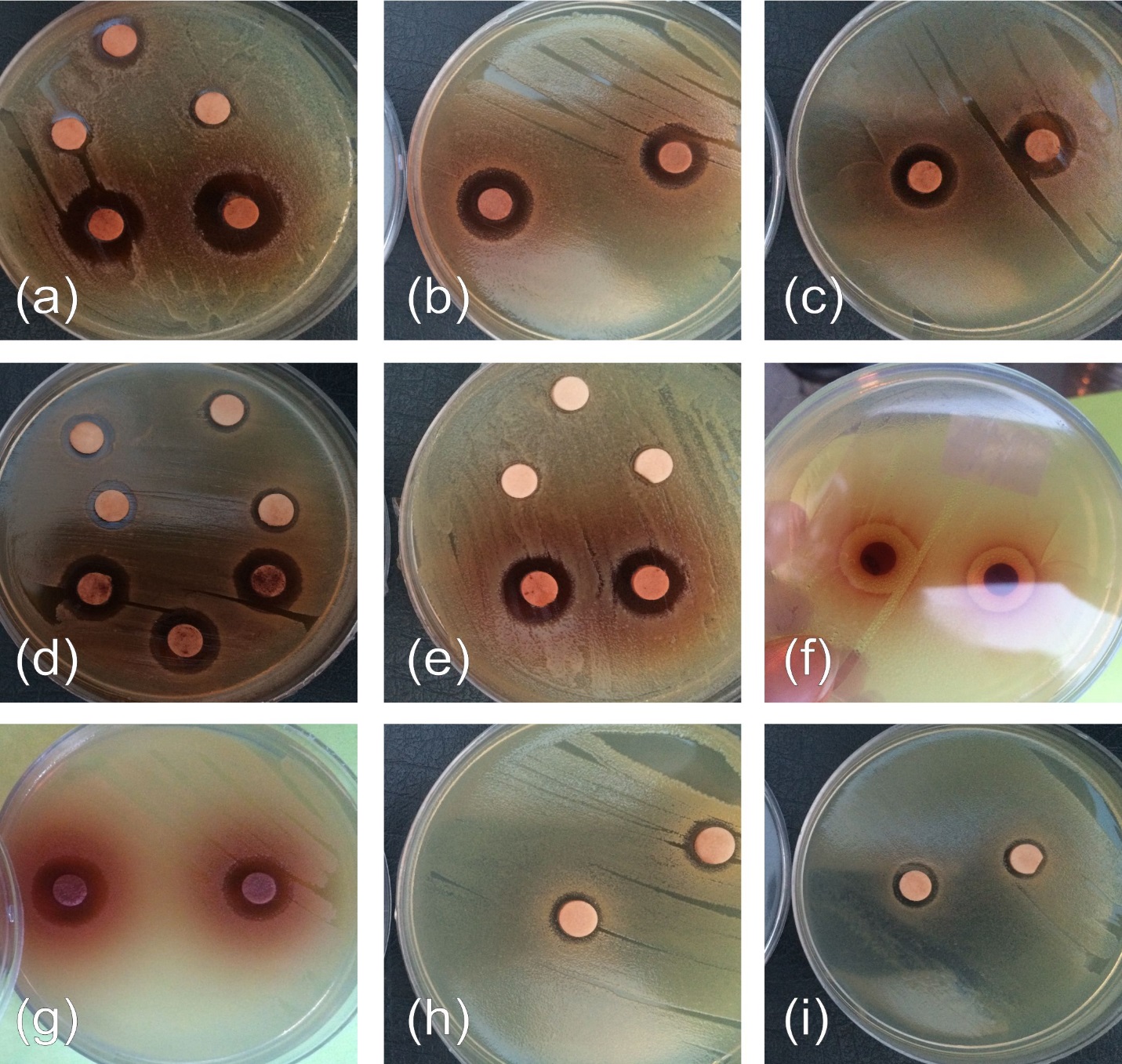
**

Fig.1s. Inhibitory activity of combined P.A extracts on *Staphylococcus aureus, Escherichia coli, Salmonella* and *Enterobacter aerogenes* in various solvents by disc diffusion method, *Escherichia coli* a)Ethanolic, b)Methanolic; *Staphylococcus aureus* c)Ethanolic, d)Methanolic; *Salmonella enterica* e)Ethanolic, f)Methanolic; *Enterobacter aerogenes* g) Aqueous, h)Ethanolic, i)Methanolic
